# Narrative Review on Post-Stroke Outcomes Through Recognition of Frailty, Sarcopenia, and Palliative Care Needs

**DOI:** 10.3390/healthcare13233011

**Published:** 2025-11-21

**Authors:** Dariusz Kotlega, Katarzyna Kobus-Kotlega, Malgorzata Szczuko

**Affiliations:** 1 Department of Pharmacology and Toxicology, University of Zielona Gora, 65-001 Zielona Gora, Poland; 2 University Hospitals of Northamptonshire—Northampton General Hospital NHS Trust, Northampton NN1 5BD, UK; 3 Neurodent Medical Centre, 65-001 Zielona Gora, Poland; 4 Department of Bromatology and Nutritional Diagnostics, Pomeranian Medical University in Szczecin, 71-460 Szczecin, Poland; malgorzata.szczuko@pum.edu.pl

**Keywords:** stroke survivors, post-stroke fatigue, falls, fear of fall, sarcopenia, palliative care, telerehabilitation, cost-effectiveness, end-of-life, disability, telemedicine, telehealth

## Abstract

Stroke survivors frequently experience long-term disability, post-stroke fatigue, frailty, sarcopenia, falls, and psychosocial distress, which together drive poorer functional recovery, rehospitalization, institutionalization, and caregiver burden. This narrative review synthesizes contemporary evidence on the prevalence, mechanisms, and clinical impact of post-stroke fatigue, frailty, sarcopenia, and falls and examines their links with palliative care needs, healthcare costs, and emerging telehealth models. A PubMed and Google Scholar search up to October 2025 identified studies on stroke and fatigue, frailty, sarcopenia, falls, palliative care, and telehealth, with an emphasis on clinical studies, trials, systematic reviews, and guidelines in adults. Frailty and sarcopenia are highly prevalent after stroke and predict mortality, poor functional outcome, reduced rehabilitation response, and higher care needs. Post-stroke fatigue is common, multifactorial, and associated with worse quality of life and reduced return-to-work rates. Falls are frequent and arise from the combined effects of focal neurological deficits and systemic frailty/sarcopenia. Despite substantial symptom burden, palliative care is often introduced late and inconsistently. We summarize brief, validated screening tools, such as the Clinical Frailty Scale, SARC-F plus grip strength, Malnutrition Universal Screening Tool, Fatigue Severity Scale/Neurological Fatigue Index for Stroke, Short Physical Performance Battery, and fall-risk instruments. We propose pragmatic timepoints and referral thresholds for their use in stroke services. Multicomponent interventions that integrate exercise, nutritional optimization, psychosocial support, and structured fall prevention can reduce frailty, sarcopenia, and falls and improve function and mood. Telemedicine and telerehabilitation may enhance access and continuity but risk widening digital inequities. Earlier, structured palliative approaches aligned with patient goals are needed across the frailty–sarcopenia–stroke continuum. Implementing integrated screening–intervention pathways and hybrid telehealth models could improve long-term outcomes for stroke survivors and their caregivers while supporting more efficient use of healthcare resources.

## 1. Introduction

Stroke is the second leading cause of death and the third leading cause of combined mortality and disability worldwide. Its global incidence is estimated at 142–151 per 100,000 population. Ischemic stroke (IS) accounts for 65.3% of cases, followed by intracerebral hemorrhage (28.8%) and subarachnoid hemorrhage (5.8%). There is an increasing rate of young patients (aged 35–44) presenting with stroke symptoms [1].

Approximately 84% of the global stroke burden is attributable to 23 modifiable risk factors, primarily metabolic (68.8%), but also environmental (36.7%) and behavioral (35.2%). Since 1990, stroke-related mortality has increased by 44%, with further rises anticipated [2,3]. Post-stroke disability affects the majority of survivors and commonly impairs activities of daily living (ADLs), motor function, cognition, and sensory domains. At six months after discharge, up to 80% of survivors demonstrate reduced ADL, 48% experience spasticity, and 34% report persistent pain; additional sequelae include new visual deficits (60%), hearing impairment (66%), cognitive dysfunction (52%), sleep disturbance (72%), and falls (60%) [4]. Significant differences exist between countries in both fatality and symptom profiles. For example, stroke-related case fatality in Poland (23.9%) was higher than in the United States (7.5%). Frequencies of several symptoms were also greater in Poland: hemiplegia (19% vs. 11%), consciousness disturbances (39% vs. 13%), dysphagia (28% vs. 14%), and aphasia (42% vs. 26%) [5]. These national and regional variations emphasize the need to adapt screening and care pathways to local population characteristics and health-system capabilities.

Long-term outcomes remain poor. In a 15-year cohort study, only 21% of patients survived; among them, 33.8% had mild disability, 14.3% had moderate disability, and 15% had severe disability, resulting in over 60% living with some degree of disability. Survivors reported a greater decline in physical than in mental quality of life [6]. The extensive care needs of stroke survivors impose a substantial burden on informal caregivers, who often face prolonged caregiving hours, increased anxiety, disrupted sleep, and financial strain [7].

This manuscript synthesizes evidence across related domains such as frailty, sarcopenia, and palliative care and presents practical screening tools and pathways for outpatient stroke services. We also propose an implementation-focused research agenda and discuss the roles of telerehabilitation, telemedicine, and cost-effectiveness analyses to improve quality and access to post-stroke services. Addressing fatigue, frailty, sarcopenia, and psychosocial distress is essential to promote neuroplasticity and maximize rehabilitation outcomes.

The rationale and objectives for this manuscript were to address the long-term phase of stroke management because it remains fragmented, with frailty, sarcopenia, malnutrition, fatigue, and falls often addressed in parallel rather than through a unified, person-centered pathway. Under-recognition of these geriatric syndromes contributes to poorer functional recovery, higher readmissions, and caregiver burden, while referrals to palliative care are typically delayed to the terminal phase despite substantial unmet symptom needs. Existing guidance is limited, screening practices vary, and there is limited practical advice on which brief tools to use, when to administer them, and how to link positive screens to specific, resource-aware interventions. At the same time, telemedicine and telerehabilitation offer opportunities to expand access and continuity of care but face barriers. This narrative review synthesizes evidence on the prevalence, mechanisms, and clinical impact of post-stroke fatigue, frailty, sarcopenia, and falls. It presents brief, validated screening instruments suitable for stroke services and proposes pragmatic timing for their use and links screening results to targeted, multidisciplinary interventions (exercise, nutritional optimization, psychosocial support). We also outline practical triggers and pathways for earlier integration of palliative approaches, including end-of-life planning where appropriate. The emerging tools, such as telemedicine/telerehabilitation, are addressed with the analysis of their outcomes, barriers, and cost-effectiveness.

## 2. Methods

This narrative review is based on the literature published until October 2025. Searches were performed in PubMed using combinations of the terms: stroke AND fatigue, stroke AND frailty, stroke AND sarcopenia, post-stroke AND falls, stroke AND telehealth, stroke AND palliative care. Preference was given to full-text articles as follows: clinical study, clinical trial, guideline, meta-analysis, randomized controlled trial, systematic review, and review. Filters included articles written in English, providing data from adults and humans. Preprints were excluded from the searches. In total, 1071 articles were selected, and, in addition, a hand search for appropriate articles in Google Scholar was performed. References were selected for relevance, with emphasis on studies addressing the integration of physical, nutritional, and psychosocial domains in stroke survivors. Eventually, 167 articles were included in this narrative review, and we present the results structured by the topics relevant to clinical practice.

## 3. Results

### 3.1. Fatigue

Post-stroke fatigue (PSF) is a frequent and clinically important sequelae of stroke, affecting 42–53% of survivors and, in some cohorts, rising to 74% at one year and 58% at three years [8,9]. Interestingly, it also affects stroke survivors with mild stroke [10].

The pathophysiology of post-stroke fatigue is multifactorial. Proposed mechanisms include direct effects of brain injury on networks regulating arousal and motivation, neuroendocrine dysregulation, systemic inflammatory responses, deconditioning, and consequences of reduced physical activity. Vascular risk factors and cerebrovascular disease burden may interact with age-related changes, premorbid frailty, and psychological factors. Self-reported visual symptoms are more frequent in PSF [11]. No neuroimaging pattern after stroke was related to PSF [12]. The presence of PSF is associated with worse functional outcomes and longer hospital stays [13]. It also affects the ability to return to work after a stroke [14].

Early identification and active management should be integral to post-stroke care. PSF is characterized by a persistent lack of energy, rapid exhaustion on physical or mental effort, and poor recovery despite rest. Multiple psychosocial, biological, and behavioral factors contribute to its development. Fatigue may present as a dominant symptom on its own or coexist with mood disorders, sleep disturbance, pain, and cognitive impairment. Clinically, PSF is associated with impaired activities of daily living and reduced health-related quality of life. The routine use of validated screening instruments, such as the Fatigue Severity Scale (FSS) and disease-specific tools such as the Neurological Fatigue Index for Stroke (NFI-Stroke), facilitates early identification of suitable patients. Comprehensive assessment should also include medication review, screening for sleep apnea and other sleep disorders, evaluation for mood disorders, and investigation of medical causes potentially related to fatigue, such as anemia, thyroid dysfunction, and infections. Early recognition of these factors is central to fatigue management plans and psychosocial support [8,9]. There are certain interventions proven to be effective in reducing post-stroke fatigue. They include physical therapy, a rehabilitation program of both conventional and robotic-assisted rehabilitation, brain stimulation with the use of transcranial direct current stimulation (tDCS), remote ischemic conditioning with the use of cuff inflation, acupuncture, naturalistic artificial sunlight lighting, education program, and supplementation of minerals and vitamins. On the other hand, psychological therapies or pharmacological interventions other than modafinil were not effective. Each kilogram of body weight gained during the first year after stroke is associated with a reduction in PSF intensity [15,16,17,18,19,20,21,22,23,24,25,26]. Weak associations were observed between impaired fitness and PSF, as well as the exercise training program promoting better sleep and reducing PSF [27,28].

We propose comprehensive support for patients identified as having moderate-to-severe fatigue. This can include a neurology/stroke physician, clinical psychologist/neuropsychologist, physiotherapist, occupational therapist, and dietitian.

### 3.2. Frailty

Frailty is a syndrome of age-related decline in physiological reserve that increases vulnerability to stressors. Clinically, frailty is defined by the presence of at least three of five criteria: low grip strength, self-reported exhaustion, slowed gait speed, low physical activity, and unintentional weight loss [29]. Risk factors associated with frailty are depression, loneliness, limitations in activities of daily living, risk of malnutrition, Dietary Inflammatory Index score, maximal walking speed, and masticatory dysfunction [30]. Contemporary instruments broaden assessment to include comorbidities, cognitive impairment, psychosocial factors, and geriatric syndromes (for example, delirium, urinary incontinence, and falls) [31]. Separate identity was discussed as ‘brain frailty’, which refers to atrophy and chronic vascular changes detected on CT or MRI scans. The presence of such a clinical syndrome is related to worse outcomes after intravenous thrombolysis or mechanical thrombectomy [32,33]. The Clinical Frailty Scale (CFS) remains widely used because its nine categories (from “very fit” to “terminally ill”) show a stepwise increase in medium-term risks of mortality and institutionalization with each one-point increment [34,35]. In community-dwelling adults aged ≥65 years, frailty prevalence is approximately 10.7%, with a further 41.6% classified as prefrail [36]. Among stroke survivors, frailty affects 22% and prefrailty about 49% [37,38]. According to a recent meta-analysis, it can even be found in 11.2 to 75.3% of stroke survivors [39]. Greater frailty severity is associated with poorer acute and long-term outcomes after stroke. Frailty occurs in 23% of people with stroke, increasing the risk of mortality (OR 2.66) and poor functional outcome (OR 2.04) [40,41]. It is related to reduced quality of life and the area of penumbra, which indicates the amount of the brain affected by stroke [42,43]. Increased complication frequency after carotid artery revascularization is observed in frail patients, but some authors suggest that its negative impact is not significant in carotid artery stenting, which might be an alternative in frail stroke survivors [44,45]. Frail patients derive less benefit from reperfusion therapy, experience higher mortality, and have worse functional outcomes at 90 days after mechanical thrombectomy. Frailty is also linked to increased complications, higher peri-procedural mortality, and greater readmission rates following carotid endarterectomy [46,47,48]. Beyond procedural risk, advanced frailty predicts a higher likelihood of post-stroke cognitive impairment and lower health-related quality of life. These relationships make frailty an important prognostic indicator in stroke medicine and a practical tool for individualizing care intensity and realistic goal setting [49].

Pre-stroke frailty status should inform discharge planning and rehabilitation pathways. Frail patients are substantially more likely to require institutional care than prefrail or non-frail survivors (46.9%, 28%, and 18.5%, respectively). Moreover, non-frail patients more often receive inpatient rehabilitation [50]. While general psychosocial or community interventions alone have a limited impact in some frail populations, structured interdisciplinary case management has demonstrated improvements in mental health and activities of daily living. This regards care coordinators, nurse care managers, therapists, and specialist stroke nurses [51,52,53]. Importantly, multicomponent exercise programs that combine balance, aerobic, and resistance training can reduce frailty even among institutionalized older adults and should be considered a core component of rehabilitation for frail survivors of stroke [54]. It has been shown that a multicomponent program including exercise, cognitive stimulation, protein supplementation, and iron replacement might be beneficial in achieving short-term improvement in their frailty status [55].

The optimal suggested interventions that could slow down frailty progression include components as follows: exercise (resistance, aerobic, and balance training), nutrition (Mediterranean diet and protein supplementation of 1.2 g/kg/day), cognitive training, psychosocial (group cognitive–behavioral therapy and social activities), pharmacologic (medication review and testosterone supplementation), multimodal (combined exercise, nutrition, and cognitive training) [56].

Given their overlapping biological underpinnings, frailty and sarcopenia frequently coexist, jointly amplifying vulnerability to fatigue, falls, and palliative needs.

### 3.3. Sarcopenia

Sarcopenia represents a loss of skeletal muscle mass and function and is closely linked to frailty, aging, disability, and multimorbidity. According to the European Working Group on Sarcopenia in Older People (EWGSOP), there are three main criteria for the definition of sarcopenia: low muscle strength, low muscle quantity or quality, and low physical performance. Fulfilling criterion 1 enables diagnosis of probable sarcopenia, while severe sarcopenia can be diagnosed when three criteria are confirmed [57].

The presence of sarcopenia is associated with a higher NIHSS score and unfavorable functional outcome in patients with acute ischemic stroke and is a predictor of poorer outcomes in muscle strength, balance, ambulation, and activities of daily living after the subacute rehabilitation program [58,59,60]. Sarcopenia has been shown to be a predictor of readmission within 6 months and poor functional outcome after stroke [61,62,63].

In stroke survivors, sarcopenia may be primary (age-related) or secondary to the neurological injury and its consequences. Post-stroke sarcopenia affects 14–18% of patients [64]. Paretic limbs commonly lose substantial muscle volume (reported declines of approximately 20–24%) as a consequence of denervation, reduced corticospinal input, and α-motoneuron loss [65]. The non-paretic side may also undergo muscle wasting related to global disuse, dysphagia (reported in up to 82.2%), anorexia, access limitations, depression, and cognitive impairment [66]. Acute and chronic stroke-related stress further promotes catabolism via sympathetic activation [67]. These neuromuscular changes reduce gait stability, diminish propulsive force, and impair reactive balance, thereby increasing fall risk and hindering functional recovery after stroke. Risk factors for stroke-related sarcopenia identified in a meta-analysis include older age, need for tube feeding, pre-existing sarcopenia, atrial fibrillation, higher initial stroke severity, and comorbid osteoporosis. Other factors, such as BMI, time since stroke, and calf circumference reduction, did not affect the risk of stroke-related sarcopenia [68]. Chronic low-grade inflammation also contributes to pre- and post-stroke sarcopenia pathogenesis [69,70]. Several molecular pathomechanisms are discussed as causes of sarcopenia: intracellular mechanisms of proteostasis and mitochondrial function, function of hormones, myo-satellite cell differentiation and proliferation, and muscle fiber composition and neuromuscular drive [71].

Malnutrition is common both at admission (≈20%) and during inpatient care (22–37%, rising to 51% in rehabilitation wards) and may pre-exist stroke or worsen thereafter [72,73]. Although the Malnutrition Universal Screening Tool (MUST) and Nutritional Risk Screening-2002 are recommended by ESPEN, few studies have applied them in stroke populations [74]. Early dysphagia screening is essential to preserve safe oral intake and reduce aspiration risk [75]. Recommended assessment methods for sarcopenia include bioelectrical impedance analysis, appendicular skeletal muscle mass (ASM) analysis based on the dual-energy X-ray absorptiometry (DEXA), CT, MRI, handgrip and pinch strength, 400 m walk test, sit-to-stand test, Short Physical Performance Battery (SPPB), Rivermead Motor Assessment, Charlson Comorbidity Index, and serum albumin/protein levels [66]. The combined method of appendicular skeletal muscle mass (ASM) with handgrip strength was also suggested [57]. Moreover, data obtained from routine neuroimaging, such as temporalis muscle thickness (TMT) and area (TMA), could be easily available indicators of sarcopenia in patients with stroke [76].

Management of sarcopenia after stroke should be multimodal and initiated early. Progressive resistance training, tailored to the individual’s neurological deficits and tolerance, is the most effective intervention to increase muscle strength and function [77]. Importantly, a non-hemiplegic side training program has also shown beneficial effects on mitigating muscle atrophy in stroke survivors, which might be a helpful guide for therapists [78]. Nutritional optimization is equally important: ensure adequate energy intake and target an appropriate daily protein intake (generally in the range of 0.8–1.2 g/kg/day, adjusted for those with marked catabolism or severe sarcopenia) [79]. Combined interventions of exercise program and medium-chain triglycerides were effective in improving the functional outcome and muscle health after stroke [80]. Leucine-enriched essential amino acid supplements improve composition and muscle strength after stroke, without affecting kidney function [81]. Certain probiotic supplementation has been shown to improve the handgrip strength in older adults [82]. Oral health including dental assessment should be incorporated into post-stroke follow-up because this aspect also affects the risk of sarcopenia. Oral health includes voice, lips, mucous membranes, tongue, gums, teeth, dentures, saliva production, and swallowing function [83].

To facilitate practical, clinical use of frailty- and sarcopenia-related problems in stroke services, we present a compact bundle of validated instruments (Table 1). The Clinical Frailty Scale (CFS) is recommended as a rapid bedside frailty screen because it is simple to apply at admission or discharge and correlates with medium-term risks of mortality and institutionalization [37,84]. This screen may also be part of a comprehensive geriatric assessment (CGA), which addresses composite geriatric needs and has beneficial effects in primary and secondary stroke therapeutic interventions [85]. The SARC-F (strength, assistance with walking, rise from a chair, climb stairs, and falls) questionnaire combined with handgrip strength provides a brief, feasible sarcopenia screen that can be performed by ward staff or community stroke team [64]. Nutritional risk should be assessed routinely using the Malnutrition Universal Screening Tool (MUST) because malnutrition is common after stroke and is a modifiable contributor to sarcopenia and poor recovery [69,70]. Post-stroke fatigue is frequent and clinically important; screening using the Fatigue Severity Scale (FSS) or the Neurological Fatigue Index for Stroke (NFI-Stroke) enables referral to fatigue management pathways and psychosocial support [8]. Finally, short performance measures such as gait speed, sit-to-stand, and the Short Physical Performance Battery (SPPB) capture functional performance and fall risk and are useful for rehabilitation planning and monitoring of response to interventions [79].

We recommend administration at three pragmatic timepoints: pre-discharge, a 3-month follow-up, and annual review for high-risk patients. Positive screens should prompt targeted physiotherapy, psychotherapy, and nutritional input. Referral triggers shown in Table 1 are intentionally conservative (e.g., CFS ≥ 5; SARC-F ≥ 4) to prioritize early multidisciplinary input while minimizing false positives in resource-constrained settings. Sarcopenia is a significant factor contributing to the risk of falls, and proactive screening and interventions can reduce the occurrence of falls in stroke survivors.

### 3.4. Fall Risk

Frailty and sarcopenia markedly increase the risk of falls. They occur in 6.7–44% of frail older adults and 10–52% of prefrail individuals [86]. In community-dwelling adults ≥65 years, 30% fall annually (50% recurrently), rising to 40% in those >85 years. Among individuals aged ≥65 years, one out of five falls leads to serious injuries, including traumatic brain injury and fractures. Key risk factors include impaired balance, prior falls, visual deficits, muscle weakness, polypharmacy (especially psychoactive medications), gait difficulties, orthostatic hypotension, depression, dizziness, advanced age, immobility, deconditioning, female sex, cognitive impairment, incontinence, diabetes, arthritis, fear of falling, and poor nutrition [87].

Post-stroke, fall incidence ranges from 7% in the first week to 37% at six months and 73% at one year [88]. Principal risk factors in this population are impaired balance and mobility, psychotropic medication use, depression, cognitive deficits, self-care disability, and prior falls [89]. Fear of falling affects up to 80% of stroke survivors and further limits activity. Therefore, targeted supportive interventions, combining psychological therapy with balance training, may reduce the risk of falls [90,91].

Post-stroke falls arise from two overlapping pathways. The first is stroke-specific neurological injury (for example, hemiplegia, ataxia, impaired proprioception, and visual field loss) that produces asymmetry, impaired motor control, and reduced reactive balance. The second is systemic sarcopenia and frailty, which reduce muscle strength, endurance, and physiological reserve. They compromise the capacity to prevent or recover from a loss of balance. Because these mechanisms commonly coexist in the same patient, clinical assessment should address both domains: detailed neurological assessment of gait, balance, and motor control, together with sarcopenia-focused measures such as handgrip strength, SARC-F, gait speed, and sit-to-stand test [68,92,93]. Clinically useful screening and assessment tools include the Falls Risk Assessment Tool (FRAT) for initial stratification, the short version of Falls Efficacy Scale-International (FES-I) to quantify fear of falling, and performance tests such as the Five-Chair Stand Test, Five Times Sit-to-Stand Test (FCST) and Timed Up and Go (TUG) to measure functional mobility [94,95,96,97]. Recommended screening instruments for sarcopenia, frailty, and fall risk are presented in Table 1. The suggested intervals represent optimal targets for well-resourced settings. In healthcare systems with constrained resources, screening frequency may be adapted according to local capacity and patient risk stratification. The cut-off points of diagnosing sarcopenia for men and women are as follows: grip strength (<27 kg, <16 kg), chair stand (>15 s), ASM (<20 kg, <15 kg), ASM/height^2^ (<7 kg/m^2^, <5.5 kg/m^2^), gait speed ≤0.8 m/s, SPPB ≤ 8 points, TUG ≥ 20 s, and 400 m walk test (non-completion or ≥6 min for completion) [57].

Interventions that contribute to reducing the risk of falls include exercise programs (such as balance training, pedaling, treadmill), housing adjustments, medication optimization, vitamin D supplementation, and nutrition [98,99,100,101]. Multifactorial interventions that address modifiable contributors, such as medication review, correction of vision problems, targeted balance and strength training, home hazard modification, feet and footwear adjustments, and management of orthostatic hypotension, reduce fall risk and are cost-effective. Rehabilitation programs that combine cognitive–behavioral approaches to address fear of falling with task-oriented balance training show benefit in both reducing fear and decreasing fall incidence [90,91]. The structured fall prevention programs are adopted by health professionals in up to 87% and patients’ adherence varies between 7% and 73% [102].

### 3.5. Palliative Care

Palliative needs in stroke survivors often emerge as downstream consequences of frailty and sarcopenia, which together reduce physiological reserve and resilience. Stroke is associated with substantial mortality both in the acute phase (6.8%) and after discharge (15.7% at one year) [103]. Fewer than half of survivors return home without additional services, and many develop sarcopenia and frailty, and more than 50% require ongoing support and palliative assessment. Despite this high need, referrals to specialist palliative care remain exceptionally low (reported 0.4–8.9%) and are typically restricted to the terminal phase. Only about half of patients die in their preferred place and 25% die in hospital [103]. In the terminal phase of stroke, up to 56% of patients are seen by palliative care services, but it varies between sites and is usually provided to less than half of such patients. More patients might need palliative care assessment to optimize care in their final days of life [104,105].

Healthcare professionals describe multiple challenges to delivering palliative care after stroke. These include difficulties maintaining consistent, clear communication, DNA CPR decisions, resolving conflicts among family members, managing unrealistic expectations, navigating prognostic uncertainty, and making decisions about “comfort” feeding for patients at risk of aspiration. Shared decision-making is therefore essential to support patients and families through complex and often unpredictable clinical courses [106,107]. The American Heart Association’s 2025 scientific statement stresses the need for a comprehensive, multidimensional assessment of distress in patients with serious neurological illness. The guidance highlights assessment domains that should include physical (pain, dyspnea, dysphagia, fatigue, seizures, aphasia), emotional (grief, anxiety, depression, agitation, psychosis), psychosocial (surrogate decision-making, financial concerns, social stigma), and spiritual needs (loss of meaning and hopelessness). It recommends that caregivers’ needs should be assessed alongside patients’ needs [108]. In the last one to two years of life, people with stroke commonly experience symptoms such as pain, dyspnea, delirium, mood changes, respiratory secretions, dysphagia, pressure ulcers, incontinence, nausea, vomiting, sleep disturbances, fatigue, communication limitations, and unintended weight loss [109]. There could be different patterns of pain observed in this group of patients, including central post-stroke pain (CPSP). It results from somatosensory tract damage and its prevalence in all patients with stroke is up to 11% and up to 50% in patients with thalamic or medullary stroke. Such neuropathic pain needs treatment adjusted to the pathomechanism, including antiepileptics [110].

Palliative care delivered alongside standard care by an interdisciplinary team aims to alleviate suffering and improve quality of life for patients with serious illnesses, including stroke. Key principles include accurate and honest prognostication, goal-concordant care planning, systematic symptom management, and support for end-of-life decision-making [111]. Practical frameworks and tools assist clinicians in identifying patients with uncertain recovery trajectories who may benefit from proactive end-of-life planning. It includes the end-of-life (EOL) pathways such as the Gold Standards Framework, Supportive and Palliative Care Indicators Tool (SPICT), and AMBER Care Bundle [112]. The AMBER Care Bundle and Dying Person’s Care Plan, introduced in 2010 for patients with limited recovery prospects, promote dignity, structured communication, shared decision-making, and preferred place of death planning. Some studies suggest that these approaches may reduce unplanned readmissions and improve the quality of EOL care [113,114]. For patients in the final 48–72 h of life, UK NICE guidance (NG31) recommends an individualized care plan that addresses anticipatory symptom control (for example, morphine, midazolam, haloperidol, hyoscine, and glycopyrronium, where appropriate), as well as discussion of artificial hydration and nutrition in line with patient and family preferences [115]. Hospitalized patients with stroke frequently experience multiple distressing symptoms that are amenable to palliative approaches. The reported prevalences include fatigue (83%), drowsiness (58%), anorexia (57%), delirium (50%), pain (36%), breathlessness (26%), respiratory secretions (26%), anxiety (24%), constipation (13%), nausea (10%), and depression (8%). Psychological distress, including low mood, loneliness, and anxiety, is experienced in almost half of stroke survivors. Symptoms that are present in more than 50% of patients are pain, incontinence, sleeplessness, confusion, and low mood. Many of these symptoms can be effectively managed within a palliative care framework [116,117,118].

Finally, research priorities should include evaluating models of integrated palliative–stroke care (for example, early specialist palliative consultation versus usual care), testing scalable education interventions for clinicians and exploring patients’ and families’ experiences of prognostic uncertainty and decision-making about artificial nutrition and preferred place of care or death. These studies should include both clinical outcomes and measures of acceptability, caregiver burden, and cost-effectiveness to inform feasible, sustainable service redesign.

The palliative care needs after stroke are the final stage of the sarcopenia–frailty–stroke continuum. Utilizing these services is crucial for improving the quality of life for survivors of stroke. It also reduces the hospital burden by avoiding unsuitable hospitalizations. We recommend that healthcare systems include these needs as an important aspect of a dignified final stage of life. We also encourage improvement in home-based access to palliative care services, as it can reduce the burden on inpatient health services [119]. There is a need for implementation of guidance and education for healthcare professionals to improve the quality and consistency of palliative care in stroke survivors [120].

### 3.6. Healthcare and Caregiver Burden

The multifaceted care needs of stroke survivors impose substantial economic strain on health and social care systems. Direct costs encompass acute-phase treatment, rehabilitation services, and secondary prevention, while long-term expenditures include social care, informal caregiving, and lost productivity. In Europe, the mean cost per stroke survivor is estimated at EUR 61,665 in the first year, including the costs of acute-phase treatment, and EUR 33,648 annually thereafter. Higher figures are reported in high-income countries [121,122]. The healthcare-related costs include rehospitalization, inpatient rehabilitation, outpatient rehabilitation, home-based rehabilitation, ambulance services, primary and specialist care, allied health services, respite care, medical tests, and medications. Non-healthcare-related costs include community services, special equipment and aids, home modification, nursing home care, informal caregiving, and employment changes. Other societal factors include the quality of life of stroke survivors and the burden on caregivers. Caregiver burden constitutes a significant part of the indirect costs of delivering care to stroke survivors, generating direct financial consequences estimated at EUR 10,508 per patient per year [122]. Moreover, caregivers experience a moderate-to-severe burden, depending on the severity of stroke-related symptoms and the level of healthcare system support available in each country [123]. Psychological distress is even more pronounced than physical strain, with depression occurring in up to 65.6% of caregivers. The global caregiver burden is further aggravated by multiple comorbidities, severe neurological impairment, dependence in daily life, aphasia, depression, low household income, provision of care exceeding 6 h per day, and caring periods longer than 3 months [124,125,126].

The occurrence of falls is another factor that increases both caregiver and healthcare resource utilization. Stroke survivors are more likely to experience falls and injurious falls than individuals without stroke, leading to higher costs and greater informal caregiver involvement [127,128]. In 2022, the average cost per fall in the United States was USD 15,212, while falls requiring hospitalization cost up to USD 34,565, depending on injury severity and length of stay [129]. Implementation of multifactorial fall prevention strategies has been shown to reduce fall risk by 26–60%. Geriatrician-led clinics applying such strategies were cost-effective for patients with a history of falls. The most common physician-led interventions include medication adjustments, referrals to other healthcare professionals, exercise programs, and lifestyle modifications [130]. The principal preventive strategies comprise structured exercise, regular vision assessment, medication review, and environmental modifications [131]. The fall prevention, exercise program “Dance to Health” implemented in England reduced the number of falls by 58% and demonstrated savings of more than GBP 196 m over a 2-year period [132].

All these factors impose major costs on healthcare systems. Implementing individualized medical follow-up for 1 and 2 years after stroke onset has been shown to be cost-effective in reducing the global burden on healthcare systems and society [133,134]. The most cost-effective post-stroke care settings were short-term inpatient units, followed by community clinics and rehabilitation centers, with subsequent community-based follow-up [135]. In Europe, shifting from center-based to home-based rehabilitation produced an additional 61,888 Quality-Adjusted Life Years (QALYs) and significant savings in healthcare and societal costs [136]. Cost-effective strategies such as home-based rehabilitation have demonstrated reductions in healthcare utilization and overall expenditure, but opposite observations were also reported [137]. Prolonging post-stroke rehabilitation until an 18-month period was not related to the functional outcomes but was cost-effective in the impact on QALYs [138]. The integrative post-stroke care can be individualized and has been shown to be cost-effective. These include stroke care coordinators, speech and language therapists, and telehealth [139]. Telemedicine, which includes telehealth and telerehabilitation interventions, can be helpful in reducing the healthcare, caregiver, and societal burden. Additional cost-effectiveness data regarding telehealth are provided in the next section.

### 3.7. Telehealth

Telemedicine represents another promising approach to improve access for patients with severe motor, visual, or cognitive impairments [140]. However, telemedicine and telerehabilitation currently lack a unified definition of service scope. They may involve a multidisciplinary team, specialized nurse coordination, internet-based guidance, real-time online sessions with a therapist, or other structured interventions [141].

Telehealth interventions such as self-care have been shown to be effective with the use of telehealth methods such as web platforms, Bluetooth devices, SMS, individual or automated phone messages, phone calls, and computer programs. Beneficial outcomes have been reported in blood pressure monitoring, diet, dysphagia evaluation, memory skills training, medication adherence, physical activity, improved motivation, and diabetes control [142,143,144,145]. A virtual multidisciplinary stroke clinic is an effective but more costly service for patients with stroke. This type of clinic included individual virtual consultations with a registered nurse, home blood pressure telemonitoring, and unlimited access to an online resource platform [146,147]. Telenursing services beneficially affect the quality of life for stroke survivors and caregivers [148]. Telehealth interventions reduce unplanned hospital admissions and could be cost-effective in elderly, frail populations [149]. Virtual reality interventions are effective in chronic, serious illnesses, including stroke, and showed benefit in reducing pain, anxiety, depression, and in improving mobility [150].

Telerehabilitation has been confirmed to be effective in improving motor function, but results are inconsistent when smartphone- or tablet-based mHealth apps, virtual or augmented reality, or technology-assisted self-rehabilitation are used. Most but not all studies have shown positive effects of telerehabilitation on functional outcome, balance, gait, cognitive functions, speech therapy, and quality of life (QoL), while evidence for benefits in activities of daily living (ADLs) is less robust. No beneficial effect on fatigue was observed when a virtual gaming program was implemented for upper limb rehabilitation. A dual-task virtual reality training program consisting of simultaneous motor and cognitive tasks was effective in reducing the risk of falls after stroke [151,152,153,154,155,156]. Moreover, telehealth services can also be useful in the virtual assessment of fall risk [157]. A supervised mHealth app program improved adherence to home exercise programs in stroke survivors, while no effect on medication adherence was observed by other authors [158,159]. The addition of virtual reality games to conventional therapy improved balance and reduced the risk of falls [160]. A recent meta-analysis compared virtual reality rehabilitation to alternative therapies and confirmed mild efficacy of virtual reality post-stroke in regard to upper limb function, balance, and activity limitation, while little or no effect was reported on gait speed [161]. A supervised telerehabilitation program consisting of three home visits, five telephone calls, and an in-home messaging device provided over three months to instruct patients in functionally based exercises and adaptive strategies showed beneficial effects on satisfaction of care but no effect on fall-related self-efficacy [162]. Reported adherence and satisfaction rates are generally high. Nevertheless, findings on the cost-effectiveness of telerehabilitation remain limited and conflicting [163]. A systematic review underscores the lack of unified trials addressing the telerehabilitation outcomes, but low-quality evidence suggests that its effectiveness is similar to in-person [164]. Other authors reported cost-effectiveness of a telehealth blood pressure monitoring program in stroke survivors [165]. A recent meta-analysis assessing the safety of telerehabilitation, though not specific to stroke, found it to be safe, with only rare, mild, and non-serious adverse events [166]. In a recent RCT, assessing the multidisciplinary virtual stroke care clinic in addition to usual care has shown that it is effective in reducing emergency admission and days of hospitalization but costs more compared to usual care alone [146].

Disparities in post-stroke rehabilitation access have been shown. This is caused by complex socioeconomic and demographic factors, such as age, gender, race and ethnicity, education level, income, insurance, geography, social support, and housing stability. Wider use of digital rehabilitation has the potential to increase access, especially for vulnerable groups [167].

Both telerehabilitation and telemedicine for multidisciplinary outpatient post-stroke care models face limitations due to patient- and staff-related barriers. Stroke survivors may be unable to independently follow telehealth guidance because of neurological impairments or may lack access to, or familiarity with, digital technologies. In addition to limited digital literacy, the patient-related barriers also include cognitive challenges, infrastructural deficits, privacy concerns, and lack of motivation due to high satisfaction with existing care. Staff-related barriers include the inability to perform comprehensive physical assessments remotely. Telehealth facilitators should be trained in digital technologies and recognize patients’ telehealth needs and challenges, as the engagement of providers affects patients’ motivation. According to a recent systematic review, patients’ barriers limiting the implementation of telehealth interventions mainly include health-related issues and patient acceptability, while the telehealth provider barriers include infrastructure challenges, time constraints, and support system deficiencies. Many of these limitations can be mitigated through the involvement of a caregiver, either a family member or a trained professional, who can assist during telemedicine sessions. Nevertheless, for many stroke survivors, telemedicine provides a substantial advantage compared with delayed or unavailable in-person care [140,168,169]. Moreover, although telemedicine is often promoted as a way to reduce disparities, evidence shows that it can paradoxically deepen inequities when vulnerable groups—those with a lower income, rural residence, limited language, or digital skills—are less able to engage [170]. Other authors reported that technical support and family members’ engagement are factors contributing to telerehabilitation sustainability [171]. This is why we suggest that combined telerehabilitation with the use of digital tools and personal induction delivered by a technical coordinator might be helpful in reducing disparities and barriers, as it has already been shown as effective [172]. Other authors suggest that goals of certain aspects of telemedicine might only be achievable in developed countries due to digital literacy and access to technology [173]. On the other hand, home blood pressure telemonitoring was effective in low-income minority stroke survivors [174].

## 4. Discussion

This narrative review highlights the interconnected nature of post-stroke fatigue, frailty, sarcopenia, fall risk, and palliative care needs. Although these domains are often considered separately in clinical practice, they share overlapping biological mechanisms, including neuroinflammation, metabolic dysregulation, reduced physiological reserve, and progressive deconditioning [8,9,29,57,68,86,103]. Their coexistence contributes to a downward functional trajectory in many stroke survivors, but early recognition and integrated management can modify outcomes.

Frailty and sarcopenia are highly prevalent after stroke and contribute to reduced rehabilitation response, prolonged hospitalization, increased risk of institutionalization, and higher mortality [38,39,40,41,42,43,57,58,59,60,61,62,63,64,68]. Despite this, routine screening in acute and community stroke services remains inconsistent. Simple tools such as the CFS, SARC-F questionnaire, handgrip strength, gait speed, and the SPPB offer practical means of identifying at-risk individuals at admission, discharge, and outpatient follow-up [36,37,64,79,85]. Positive screening assessments should prompt early exercise rehabilitation, nutritional optimization, and psychosocial support, which eventually lead to improvements in function, fatigue, mood, and quality of life, even among older and institutionalized patients [50,54,55,56,79,80,81,82,83]. These findings support a shift from opportunistic case finding to structured, protocolized screening across the post-stroke care.

Post-stroke fatigue remains difficult to manage due to its multifactorial nature. Evidence from observational cohorts and interventional trials suggests that PSF is driven by direct effects of brain injury, sleep disturbance, mood disorders, systemic inflammation, deconditioning, and medication effects [8,9,10,11,12,16]. No single intervention is sufficient. Physical rehabilitation, including conventional and robotic programs, non-invasive brain stimulation (tDCS), remote ischemic conditioning, acupuncture, naturalistic light exposure, education programs, and nutritional supplementation have all shown varying degrees of benefit [15,16,17,18,27,28]. In contrast, most psychological interventions alone and several pharmacological therapies other than modafinil show modest or inconsistent effects [16,17,18]. This supports the need for integrated care plans that combine physical, nutritional, and psychological components tailored to patient-specific drivers of fatigue [8,9,15,16,17,18,19,20,21,22,23,24,25,26,27,28].

Falls illustrate how stroke-specific neurological deficits and systemic frailty/sarcopenia converge. Motor asymmetry, ataxia, sensory loss, and visual field defects interact with reduced muscle mass, weakness, and impaired balance to create a high and persistent fall risk [86,87,88,89]. Post-stroke fall incidence may reach 73% at one year, and fear of falling affects a large proportion of survivors, further restricting activity [88,89,90,91]. Multifactorial interventions that combine balance and resistance training, medication review, vision correction, environmental modifications, vitamin D, or nutritional interventions reduce falls and are generally cost-effective [90,91,94,95,96,97,98,99,100,101,102]. Embedding standardized fall screening and prevention bundles into stroke pathways, with clear referral criteria to specialist fall services, is therefore an important component of secondary prevention [94,95,96,97,98,99,100,101,102].

This review also highlights a large gap between the symptom burden experienced by stroke survivors and the provision of palliative care. Many patients have complex combinations of pain, dyspnea, dysphagia, fatigue, delirium, mood disturbance, and existential distress, yet referrals to specialist palliative services remain low and often restricted to the terminal phase [103,104,105,106,107,108,109,110,111,116,117,118,119]. Frameworks such as the Gold Standards Framework, the Supportive and Palliative Care Indicators Tool (SPICT), the AMBER Care Bundle, and NICE guideline NG31 offer practical triggers for earlier identification of patients at risk of deterioration or limited recovery [112,113,114,115,116]. Integrating these tools into stroke services may support more timely goals-of-care discussions, better symptom control, and more consistent respect for preferred place of care and death [112,113,114,115,116,117,118,119,120].

Telemedicine and telerehabilitation represent important opportunities to expand access to post-stroke services, especially for patients with severe disability, rural residence, or limited transport [140,141,142,143,144,145,146,147,151]. Telehealth interventions can support blood pressure control, self-management, rehabilitation, memory rehabilitation, dysphagia assessment, and caregiver education. Virtual multidisciplinary stroke clinics have demonstrated benefits in quality of life and reduced emergency admissions, albeit at higher program costs [142,143,144,145,146,147,148,149,151,167,168]. At the same time, there are significant barriers—cognitive impairment, aphasia, limited digital literacy, technology access, staff time, and infrastructural constraints—with the potential to widen inequities if vulnerable groups are less able to participate [168,169,170,171]. Hybrid models combining in-person assessment with digitally supported follow-up and explicit involvement of family or paid caregivers may offer the most realistic and equitable implementation strategy [148,149,150,151,152,160,161,162,163,164,167,168,169].

From a health-system perspective, the long-term costs of stroke are substantial, driven by rehospitalization, institutional care, informal caregiving, and productivity loss [121,122,123,124,125,126,127,128]. Falls, in particular, add significantly to healthcare expenditure and caregiver burden, with fall-related hospitalizations among stroke survivors associated with high direct costs [127,128,129,130,131,132]. Cost-effectiveness analyses suggest that individualized follow-up, home-based or community-based rehabilitation, and integrated stroke care models (including stroke coordinators and telehealth components) can reduce overall costs while maintaining or improving outcomes [133,134,135,136,137,138,139]. Multifactorial fall prevention programs and community exercise initiatives have also demonstrated favorable economic profiles [130,131,132].

This review has several limitations. It is a narrative rather than a systematic review, which introduces potential selection and publication bias. The included studies vary in design, follow-up duration, and tools used to define frailty, sarcopenia, and fatigue, as well as in the content and intensity of interventions [8,9,29,57,86,140]. Evidence regarding telehealth and integrated palliative pathways is still evolving, and many trials are small or single-center with heterogeneous outcome measures [140,141,142,143,144,145,146,147,151,152,153,154,155,156,157,158,159,160,161,162,163,164,165]. Nonetheless, by integrating findings across multiple domains and focusing on practical tools and pathways, this review aims to provide a clinically useful framework for stroke services.

**Figure 1:**
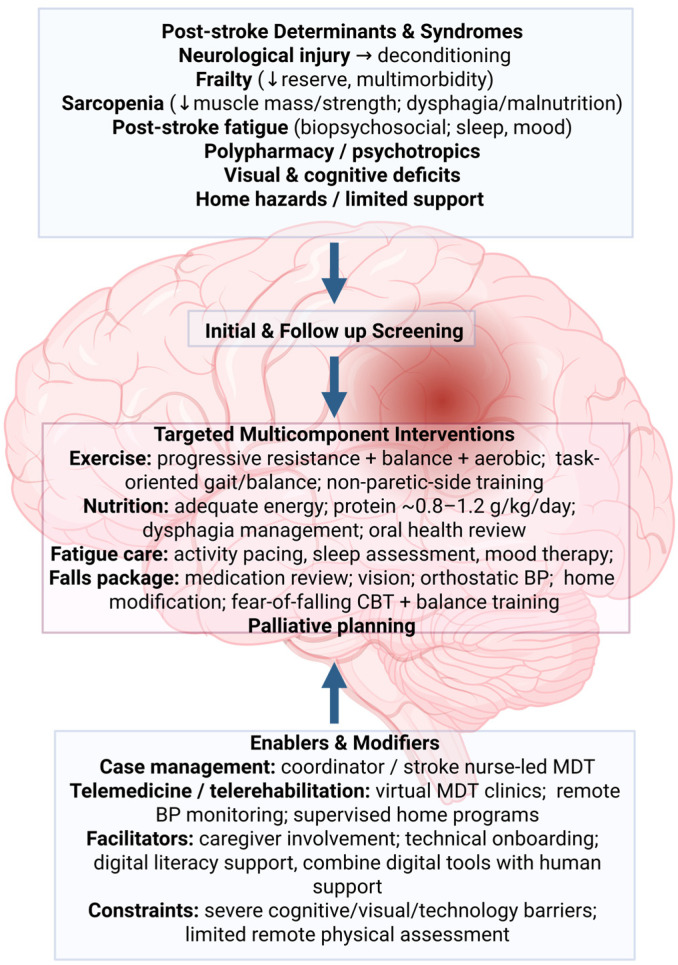
Multifactorial components and interventions in post-stroke care.

Overall, the findings support a conceptual model in which frailty, sarcopenia, post-stroke fatigue, falls, and palliative needs form a continuum across the post-stroke trajectory [38,39,40,41,42,43,57,58,59,60,61,62,63,64,86,103,119]. Early, structured screening and integrated multidisciplinary interventions have the potential to improve outcomes for patients and caregivers and to reduce the long-term burden on health and social care systems [54,55,56,79,80,81,82,83,121,122,123,124,125,126,127,128,129,130,131,132,133,134,135,136,137,138,139]. Figure 1 summarizes these interrelated components and the key intervention points along the pathway.

## 5. Conclusions

Stroke survivors frequently face a complex cluster of long-term problems, including disability, frailty, sarcopenia, fatigue, falls, and psychosocial distress. These factors are strongly associated with mortality, rehospitalization, institutionalization, and reduced quality of life. The evidence reviewed here indicates that routine use of brief, validated screening tools, combined with early, multimodal interventions, can modify the outcome.

We recommend that stroke services systematically incorporate frailty, sarcopenia, nutritional, and fall-risk screening into inpatient, outpatient, and community pathways and link positive screens to targeted exercise, nutritional, and psychosocial programs. Telemedicine and telerehabilitation should be developed as complementary, not replacement, strategies to improve access and continuity, with attention to digital literacy and equity. Palliative approaches ought to be integrated earlier for patients with high symptom burden or limited recovery potential, using structured triggers and shared decision-making to guide care.

Future research should prioritize pragmatic, multicenter trials of integrated screening–intervention packages, explicit cost-effectiveness analyses, and studies capturing caregiver outcomes and patient-reported experience. Greater methodological consistency in telehealth and post-stroke rehabilitation trials is needed to guide service redesign. By aligning frailty, sarcopenia, falls, telehealth, and palliative frameworks within unified post-stroke pathways, health systems may improve long-term outcomes for survivors while using resources more efficiently.

## Figures and Tables

**Table 1:** Recommended screening instruments for sarcopenia, frailty, and fall risk.

Instrument	Purpose	Approximate Testing Time	Action
Clinical Frailty Scale (CFS)	Rapid frailty screen	~1–2 min	CFS ≥ 5 → geriatric assessment/multidisciplinary case management; CFS ≥ 8 consider palliative assessment
SARC-F (questionnaire) + handgrip strength	Initial sarcopenia screen	3–5 min	SARC-F ≥ 4 or low grip strength → refer to physiotherapy and dietician
MUST (Malnutrition Universal Screening Tool)	Nutritional risk	2–3 min	MUST ≥ 1 → dietitian referral
Fatigue Severity Scale (FSS) or NFI-Stroke	Screen for post-stroke fatigue	5–7 min	High score → fatigue management pathway (psychology, sleep assessment, activity pacing)
Short Physical Performance Battery (SPPB)/gait speed/sit-to-stand	Functional performance and fall risk	5–10 min	Low performance/gait speed → targeted physiotherapy, Falls clinic
Falls Risk Assessment Tool (FRAT)	Fall risk	5–10 min	Moderate or high risk → Falls clinic
Falls Efficacy Scale-International short version (FES-I)	Fall risk	2–5 min	Moderate or high risk → Falls clinic
Five-Chair Stand Test, Five Times Sit-to-Stand Test (FCST)	Fall risk	2–5 min	≥15 s → Falls clinic
Timed Up and Go (TUG)	Fall risk	2 min	≥15 s → Falls clinic

## Data Availability

No new data were created or analyzed in this study.

## References

[1] Jermakow N., Maluchnik M., Sienkiewicz-Jarosz H., Karaszewski B., Wierzchowska-Cioch E., Ryglewicz D. (2022). Trends of Stroke Hospitalisation and Fatality Rates in Young vs. Elderly People in Poland during 2010–2019 Decade. Neurol. Neurochir. Pol..

[2] Feigin V.L., Brainin M., Norrving B., Martins S.O., Pandian J., Lindsay P., Grupper F., Rautalin I. (2025). World Stroke Organization: Global Stroke Fact Sheet 2025. Int. J. Stroke.

[3] Fan J., Li X., Yu X., Liu Z., Jiang Y., Fang Y., Zong M., Suo C., Man Q., Xiong L. (2023). Global Burden, Risk Factor Analysis, and Prediction Study of Ischemic Stroke, 1990–2030. Neurology.

[4] Poomalai G., Prabhakar S., Sirala Jagadesh N. (2023). Functional Ability and Health Problems of Stroke Survivors: An Explorative Study. Cureus.

[5] Ryglewicz D., Hier D.B., Wiszniewska M., Cichy S., Lechowicz W., Członkowska A. (2000). Ischemic Strokes Are More Severe in Poland than in the United States. Neurology.

[6] Crichton S.L., Bray B.D., McKevitt C., Rudd A.G., Wolfe C.D.A. (2016). Patient Outcomes up to 15 Years after Stroke: Survival, Disability, Quality of Life, Cognition and Mental Health. J. Neurol. Neurosurg. Psychiatry.

[7] Kumar A., Yadav A.K., Singh V.K., Pathak A., Chaurasia R.N., Mishra V.N., Joshi D. (2022). Caregiver Burden in Caregivers of Stroke Survivors: A Hospital-Based Study. Ann. Indian Acad. Neurol..

[8] Chen W., Jiang T., Huang H., Zeng J. (2023). Post-Stroke Fatigue: A Review of Development, Prevalence, Predisposing Factors, Measurements, and Treatments. Front. Neurol..

[9] Chen Y.K., Qu J.F., Xiao W.M., Li W.Y., Weng H.Y., Li W., Liu Y.L., Luo G.P., Fang X.W., Ungvari G.S. (2015). Poststroke Fatigue: Risk Factors and Its Effect on Functional Status and Health-Related Quality of Life. Int. J. Stroke.

[10] Vitturi B.K., Mitre L.P., Kim A.I.H., Gagliardi R.J. (2021). Prevalence and Predictors of Fatigue and Neuropsychiatric Symptoms in Patients with Minor Ischemic Stroke. J. Stroke Cerebrovasc. Dis..

[11] Pedersen S.G., Løkholm M., Friborg O., Halvorsen M.B., Kirkevold M., Heiberg G., Anke A. (2023). Visual Problems Are Associated with Long-Term Fatigue after Stroke. J. Rehabil. Med..

[12] García-Bouyssou I., Laredo C., Massons M., Serrano M., Moreira F., Cabero-Arnold A., Urra X., Chamorro A. (2024). Clinical and Neuroanatomical Predictors of Post-Stroke Fatigue. J. Stroke Cerebrovasc. Dis..

[13] Zeng H., Yang J., Wu J., Ding Y., Yuan S., Wang R., Zhao W., Zeng X. (2024). The Impact of Post-Stroke Fatigue on Inpatient Rehabilitation Outcomes: An Observational Study. PLoS ONE.

[14] Rutkowski N.A., Sabri E., Yang C. (2021). Post-Stroke Fatigue: A Factor Associated with Inability to Return to Work in Patients <60 Years—A 1-Year Follow-Up. PLoS ONE.

[15] Castelli L., Giannuzzi D., Loreti C., Falcolini I., Tamburro E., Malizia A.M., Iacovelli C., Biscotti L., Padua L., Giovannini S. (2025). The Impact of Robotic Hand Rehabilitation on Hand Function and Fatigue in Patients with Stroke (RoHa-S). J. Clin. Neurosci..

[16] Giovannini S., Iacovelli C., Loreti C., Lama E., Morciano N., Frisullo G., Biscotti L., Padua L., Castelli L. (2024). The Role of Nutritional Supplement on Post-Stroke Fatigue: A Pilot Randomized Controlled Trial. J. Nutr. Health Aging.

[17] Komber A., Chu S.H., Zhao X., Komber H., Halbesma N., Mead G. (2024). Non-Pharmacological Interventions for the Treatment of Post-Stroke Fatigue: A Systematic Review. Int. J. Stroke.

[18] Moyle D.B., Kudiersky M.N., Totton M.N., Sassani D.M., Nichols D.S., Jenkins D.T., Redgrave D.J., Baig D.S., Nair D.K.P.S., Majid P.A. (2023). Remote Ischaemic Conditioning for Fatigue after Stroke (RICFAST): A Pilot Randomised Controlled Trial. J. Stroke Cerebrovasc. Dis..

[19] Chu S.H., Zhao X., Komber A., Cheyne J., Wu S., Cowey E., Kutlubaev M., Mead G. (2023). Pharmacological Interventions for the Treatment of Post-Stroke Fatigue: A Systematic Review. Int. J. Stroke.

[20] You J., Li H., Xie D., Chen M., Chen R. (2023). Efficacy of Acupuncture Therapy for Post-Stroke Fatigue: A Systematic Review and Meta-Analysis. J. Tradit. Chin. Med..

[21] Dong X.L., Sun X., Sun W.M., Yuan Q., Yu G.H., Shuai L., Yuan Y.F. (2021). A Randomized Controlled Trial to Explore the Efficacy and Safety of Transcranial Direct Current Stimulation on Patients with Post-Stroke Fatigue. Medicine.

[22] De Doncker W., Ondobaka S., Kuppuswamy A. (2021). Effect of Transcranial Direct Current Stimulation on Post-Stroke Fatigue. J. Neurol..

[23] West A., Simonsen S.A., Jennum P., Cyril Hansen N., Schønsted M., Zielinski A., Sander B., Iversen H.K. (2019). An Exploratory Investigation of the Effect of Naturalistic Light on Fatigue and Subjective Sleep Quality in Stroke Patients Admitted for Rehabilitation: A Randomized Controlled Trial. NeuroRehabilitation.

[24] Bivard A., Lillicrap T., Krishnamurthy V., Holliday E., Attia J., Pagram H., Nilsson M., Parsons M., Levi C.R. (2017). MIDAS (Modafinil in Debilitating Fatigue after Stroke): A Randomized, Double-Blind, Placebo-Controlled, Cross-Over Trial. Stroke.

[25] Delbridge A., Howlett O., English C., Simpson D.B. (2025). What Is the Effect of Education on Fatigue in Adults with Neurological Conditions? A Systematic Review and Meta-Analysis. Clin. Rehabil..

[26] Larsson P., Edvardsen E., Gay C.L., Ursin M., Ihle-Hansen H., Hagen P.M., Lerdal P.A. (2025). Changes in Fatigue after First-Ever Ischemic Stroke and Their Associations with Changes in Physical Fitness, Body Composition, and Physical Activity. J. Stroke Cerebrovasc. Dis..

[27] Larsson P., Bidonde J., Olsen U., Gay C.L., Lerdal A., Ursin M., Mead G.E., Edvardsen E. (2023). Association of Post-Stroke Fatigue with Physical Activity and Physical Fitness: A Systematic Review and Meta-Analysis. Int. J. Stroke.

[28] Tai D., Falck R.S., Davis J.C., Vint Z., Liu-Ambrose T. (2022). Can Exercise Training Promote Better Sleep and Reduced Fatigue in People with Chronic Stroke? A Systematic Review. J. Sleep. Res..

[29] Fried L.P., Tangen C.M., Walston J., Newman A.B., Hirsch C., Gottdiener J., Seeman T., Tracy R., Kop W.J., Burke G. (2001). Frailty in Older Adults: Evidence for a Phenotype. J. Gerontol. A Biol. Sci. Med. Sci..

[30] Boucham M., Salhi A., El Hajji N., Gbenonsi G.Y., Belyamani L., Khalis M. (2024). Factors Associated with Frailty in Older People: An Umbrella Review. BMC Geriatr..

[31] Xue Q.L. (2011). The Frailty Syndrome: Definition and Natural History. Clin. Geriatr. Med..

[32] Loewen S.P., Singh N., Alhabli I., Bala F., Buck B., Benali F., Betzner W., Lam K., Catanese L., Tkach A. (2025). Brain Frailty and Functional Outcomes After Thrombolysis for Acute Ischemic Stroke. JAMA Netw. Open.

[33] Chan V., Rheaume A.R., Chow M.M. (2022). Impact of Frailty on 30-Day Death, Stroke, or Myocardial Infarction in Severe Carotid Stenosis: Endarterectomy versus Stenting. Clin. Neurol. Neurosurg..

[34] Church S., Rogers E., Rockwood K., Theou O. (2020). A Scoping Review of the Clinical Frailty Scale. BMC Geriatr..

[35] Rockwood K., Song X., MacKnight C., Bergman H., Hogan D.B., McDowell I., Mitnitski A. (2005). A Global Clinical Measure of Fitness and Frailty in Elderly People. CMAJ.

[36] Collard R.M., Boter H., Schoevers R.A., Oude Voshaar R.C. (2012). Prevalence of Frailty in Community-Dwelling Older Persons: A Systematic Review. J. Am. Geriatr. Soc..

[37] Palmer K., Vetrano D.L., Padua L., Marengoni A., Bernabei R., Bruno G., Cesari M., Evans J.G., Lattanzio F., Roller-Wirnsberger R. (2019). Frailty Syndromes in Persons with Cerebrovascular Disease: A Systematic Review and Meta-Analysis. Front. Neurol..

[38] Fernandes J., Gomes C.D.S., Guerra R.O., Alvarado B.E., Zunzunegui M.V. (2021). Frailty Syndrome and Risk of Cardiovascular Disease: Analysis from the International Mobility in Aging Study. Arch. Gerontol. Geriatr..

[39] Wang S., Tan J., Song K., Zhang Q., Yang W., Wu Y. (2025). Impact of Pre-Existing Frailty on All-Cause Mortality in Stroke Survivors: A Systematic Review and Dose–Response Meta-Analysis. Age Ageing.

[40] Li J., Wan J., Wang H. (2024). Role of Frailty in Predicting Outcomes after Stroke: A Systematic Review and Meta-Analysis. Front. Psychiatry.

[41] Chen S.F., Li H.H., Guo Z.N., Ling K.Y., Yu X.L., Liu F., Zhu X.P., Zhu X. (2024). Association between Pre-Stroke Frailty Status and Stroke Risk and Impact on Outcomes: A Systematic Review and Meta-Analysis of 1,660,328 Participants. Aging Clin. Exp. Res..

[42] Dohle E., Lewis B., Agarwal S., Warburton E.A., Evans N.R. (2024). Frailty Reduces Penumbral Volumes and Attenuates Treatment Response in Hyperacute Ischemic Stroke. Age Ageing.

[43] Wæhler I.S., Saltvedt I., Lydersen S., Fure B., Askim T., Einstad M.S., Thingstad P. (2021). Association between In-Hospital Frailty and Health-Related Quality of Life after Stroke: The Nor-COAST Study. BMC Neurol..

[44] Fladt J., Benali F., Jaroenngarmsamer T., Bala F., Singh N., Nogueira R.G., McTaggart R., Demchuk A., Poppe A., Buck B.H. (2025). ESCAPE-NA1 Investigators. Impact of Brain Frailty on Clinical Presentation and Neurologic Recovery in Acute Ischemic Stroke Patients Undergoing Thrombectomy. Neurology.

[45] Liu Z., Yao Y., Zhang M., Ling Y., Yao X., Hu M. (2023). Prevalence and Adverse Outcomes of Pre-Operative Frailty in Patients Undergoing Carotid Artery Revascularization: A Meta-Analysis. Front. Cardiovasc. Med..

[46] Pandit V., Lee A., Zeeshan M., Kulvatunyou N., Hamidi M., O’Keeffe T., Rhee P., Tang A., Joseph B. (2020). Effect of Frailty Syndrome on the Outcomes of Patients with Carotid Stenosis. J. Vasc. Surg..

[47] Mandelbaum A.D., Hadaya J., Ulloa J.G., Patel R., McCallum J.C., De Virgilio C., Benharash P. (2021). Impact of Frailty on Clinical Outcomes after Carotid Artery Revascularization. Ann. Vasc. Surg..

[48] Schnieder M., Bähr M., Kirsch M., Maier I., Behme D., Riedel C.H., Psychogios M.N., Brehm A., Liman J., von Arnim C.A.F. (2021). Analysis of Frailty in Geriatric Patients as a Prognostic Factor in Endovascular Treated Patients with Large Vessel Occlusion Strokes. J. Clin. Med..

[49] Evans N.R., Todd O.M., Minhas J.S., Fearon P., Harston G.W., Mant J., Mead G., Hewitt J., Quinn T.J., Warburton E.A. (2022). Frailty and Cerebrovascular Disease: Concepts and Clinical Implications for Stroke Medicine. Int. J. Stroke.

[50] Seamon B.A., Simpson K.N. (2019). The Effect of Frailty on Discharge Location for Medicare Beneficiaries after Acute Stroke. Arch. Phys. Med. Rehabil..

[51] Ertel K.A., Glymour M.M., Glass T.A., Berkman L.F. (2007). Frailty Modifies Effectiveness of Psychosocial Intervention in Recovery from Stroke. Clin. Rehabil..

[52] Saragih I.D., Saragih I.S., Tarihoran D.E.T.A.U., Sharma S., Chou F.H. (2023). A Meta-Analysis of Studies of the Effects of Case Management Intervention for Stroke Survivors Across Three Countries. J. Nurs. Scholarsh..

[53] Camicia M., Lutz B., Summers D., Klassman L., Vaughn S. (2021). Nursing’s Role in Successful Stroke Care Transitions Across the Continuum: From Acute Care into the Community. Stroke.

[54] Martínez-Montas G.F., Sanz-Matesanz M., Benítez-Sillero J.D.D., Martínez-Aranda L.M. (2025). Prevention and Mitigation of Frailty Syndrome in Institutionalised Older Adults Through Physical Activity: A Systematic Review. Healthcare.

[55] Ahmad F., Fountotos R., Goldfarb M., Bharaj N., Munir H., Marsala J., Rudski L.G., Afilalo J. (2023). De-Frailing Intervention for Hospitalized Cardiovascular Patients in the TARGET-EFT Randomized Clinical Trial. Eur. Heart J. Qual. Care Clin. Outcomes.

[56] Fan X., Xia Y., Xu S., Jia S. (2025). A Narrative Review of Interventions for Post-Stroke Frailty: Current Advances and Future Directions. Front. Neurol..

[57] Cruz-Jentoft A.J., Bahat G., Bauer J., Boirie Y., Bruyère O., Cederholm T., Cooper C., Landi F., Rolland Y., Sayer A.A. (2019). Writing Group for the European Working Group on Sarcopenia in Older People 2 (EWGSOP2); and the Extended Group for EWGSOP2. Sarcopenia: Revised European Consensus on Definition and Diagnosis. Age Ageing.

[58] Han M., Lim I.H., Hong S.H., Nam H.S., Heo J.H., Kim Y.D. (2024). Initial Stroke Severity and Discharge Outcome in Patients with Muscle Mass Deficit. Sci. Rep..

[59] Kim S.-Y., Cho W.-S., Park C.-B., Kim B.-G. (2024). Effect of Sarcopenia on Functional Recovery in Acute Stroke Patients Admitted for Standard Rehabilitation Program. Medicina.

[60] Abe T., Iwata K., Yoshimura Y., Shinoda T., Inagaki Y., Ohya S., Yamada K., Oyanagi K., Maekawa Y., Honda A. (2020). Low Muscle Mass Is Associated with Walking Function in Patients with Acute Ischemic Stroke. J. Stroke Cerebrovasc. Dis..

[61] Chen R., Liu Z., Liao R., Liang H., Hu C., Zhang X., Chen J., Xiao H., Ye J., Guo J. (2025). The Effect of Sarcopenia on Prognosis in Patients with Mild Acute Ischemic Stroke: A Prospective Cohort Study. BMC Neurol..

[62] Lee H., Lee I.H., Heo J., Baik M., Park H., Lee H.S., Nam H.S., Kim Y.D. (2022). Impact of Sarcopenia on Functional Outcomes among Patients with Mild Acute Ischemic Stroke and Transient Ischemic Attack: A Retrospective Study. Front. Neurol..

[63] Nozoe M., Kubo H., Yamamoto M., Ikeji R., Seike H., Majima K., Shimada S. (2024). Muscle Weakness Is More Strongly Associated with Functional Outcomes in Patients with Stroke than Sarcopenia or Muscle Wasting: An Observational Study. Aging Clin. Exp. Res..

[64] Ryan A.S., Ivey F.M., Serra M.C., Macko R.F., Hafer-Macko C.E. (2017). Sarcopenia and Physical Function in Middle-Aged and Older Stroke Survivors. Arch. Phys. Med. Rehabil..

[65] Drey M., Krieger B., Sieber C.C., Bauer J.M., Hettwer S., Ball L., Miko I., Bertsch T., Sieber C.C. (2014). Motoneuron Loss Is Associated with Sarcopenia. J. Am. Med. Dir. Assoc..

[66] Li W., Yue T., Liu Y. (2020). New Understanding of the Pathogenesis and Treatment of Stroke-Related Sarcopenia. Biomed. Pharmacother..

[67] Springer J., Schust S., Peske K., Tschirner A., Mäckenzie S., Martínez A., Kluge M., Troseid M., Jansson A., Anker S.D. (2014). Catabolic Signaling and Muscle Wasting after Acute Ischemic Stroke in Mice: Indication for a Stroke-Specific Sarcopenia. Stroke.

[68] Yan H., Li J., Xian L., Li Y., Li S., Wen Q. (2025). Risk Factors of Stroke-Related Sarcopenia: A Systematic Review and Meta-Analysis. Front. Aging.

[69] Yoshimura Y., Bise T., Nagano F., Shimazu S., Shiraishi A., Yamaga M., Koga H. (2018). Systemic Inflammation in the Recovery Stage of Stroke: Its Association with Sarcopenia and Poor Functional Rehabilitation Outcomes. Prog. Rehabil. Med..

[70] Dalle S., Rossmeislova L., Koppo K. (2017). The Role of Inflammation in Age-Related Sarcopenia. Front. Physiol..

[71] Wiedmer P., Jung T., Castro J.P., Pomatto L.C.D., Sun P.Y., Davies K.J.A., Grune T. (2021). Sarcopenia—Molecular Mechanisms and Open Questions. Ageing Res. Rev..

[72] Di Vincenzo O., Luisi M.L.E., Alicante P., Ballarin G., Biffi B., Gheri C.F., Scalfi L. (2023). The Assessment of the Risk of Malnutrition (Undernutrition) in Stroke Patients. Nutrients.

[73] Sabbouh T., Torbey M.T. (2018). Malnutrition in Stroke Patients: Risk Factors, Assessment, and Management. Neurocrit. Care.

[74] Serón-Arbeloa C., Labarta-Monzón L., Puzo-Foncillas J., Mallor-Bonet T., Lafita-López A., Bueno-Vidales N., Montoro-Huguet M. (2022). Malnutrition Screening and Assessment. Nutrients.

[75] Carnaby G., Hankey G.J., Pizzi J. (2006). Behavioural Intervention for Dysphagia in Acute Stroke: A Randomised Controlled Trial. Lancet Neurol..

[76] Yang Y.C., Yin C.H., Lin P.C., Shiue Y.L. (2025). Association between Temporalis Muscle Thickness and Functional Outcomes in Acute Stroke: A Meta-Analysis and GRADE Approach. J. Nutr. Health Aging.

[77] Nascimento C.M., Ingles M., Salvador-Pascual A., Cominetti M.R., Gomez-Cabrera M.C., Viña J. (2019). Sarcopenia, Frailty and Their Prevention by Exercise. Free Radic. Biol. Med..

[78] Feng T., Zhao C., Dong J., Xue Z., Cai F., Li X., Hu Z., Xue X. (2024). The Effect of Unaffected Side Resistance Training on Upper Limb Function Reconstruction and Prevention of Sarcopenia in Stroke Patients: A Randomized Controlled Trial. Sci. Rep..

[79] Sions J.M., Tyrell C.M., Knarr B.A., Jancosko A., Binder-Macleod S.A. (2012). Age- and Stroke-Related Skeletal Muscle Changes: A Review for the Geriatric Clinician. J. Geriatr. Phys. Ther..

[80] Yoshimura Y., Nagano F., Matsumoto A., Shimazu S., Shiraishi A., Kido Y., Bise T., Hamada T., Yoneda K. (2025). Synergistic Effects of Medium-Chain Triglyceride Supplementation and Resistance Training on Physical Function and Muscle Health in Post-Stroke Patients. Nutrients.

[81] Nakagawa N., Koyama S., Maruyama K., Maruyama J.I., Hasebe N. (2024). Effects of Nutritional Support with a Leucine-Enriched Essential Amino Acid Supplement on Body Composition, Muscle Strength, and Physical Function in Stroke Patients Undergoing Rehabilitation. Nutrients.

[82] Qaisar R., Burki A., Karim A., Iqbal M.S., Ahmad F. (2024). Probiotics Supplements Improve the Sarcopenia-Related Quality of Life in Older Adults with Age-Related Muscle Decline. Calcif. Tissue Int..

[83] Shiraishi A., Yoshimura Y., Wakabayashi H., Nagano F., Matsumoto A., Shimazu S., Kido Y., Bise T., Kuzuhara A., Hori K. (2025). Impaired Oral Status Is Associated with Sarcopenic Obesity in Post-Stroke Patients. Gerodontology.

[84] Patel K., Shrier W.E.J., Sengupta N., Hunt D.C.E., Hodgson L.E. (2022). Frailty, Assessed by the Rockwood Clinical Frailty Scale and 1-Year Outcomes Following Ischaemic Stroke in a Non-Specialist UK Stroke Centre. J. Stroke Cerebrovasc. Dis..

[85] Chirap-Mitulschi I.A., Antoniu S., Schreiner T.G. (2024). The Impact of Palliative Care on the Frailty–Stroke Continuum: From Theoretical Concepts to Practical Aspects. Postgrad. Med..

[86] Fhon J.R., Rodrigues R.A., Neira W.F., Huayta V.M., Robazzi M.L. (2016). Fall and Its Association with the Frailty Syndrome in the Elderly: Systematic Review with Meta-Analysis. Rev. Esc. Enferm. USP.

[87] Appeadu M.K., Bordoni B. (2025). Falls and Fall Prevention in Older Adults. StatPearls *[Internet]*.

[88] Denissen S., Staring W., Kunkel D., Pickering R.M., Lennon S., Geurts A.C., Weerdesteyn V., Verheyden G.S. (2019). Interventions for Preventing Falls in People after Stroke. Cochrane Database Syst. Rev..

[89] Wei W.E., De Silva D.A., Chang H.M., Tan C.S., Koh G.C.H., Matchar D.B. (2019). Post-Stroke Patients with Moderate Function Have the Greatest Risk of Falls: A National Cohort Study. BMC Geriatr..

[90] Chen Y., Du H., Song M., Li Q., Liu S., Wei L., Zhang Y. (2023). Relationship between Fear of Falling and Fall Risk among Older Patients with Stroke: A Structural Equation Modeling. BMC Geriatr..

[91] Liu T.W., Ng G.Y.F., Chung R.C.K., Ng S.S.M. (2019). Decreasing Fear of Falling in Chronic Stroke Survivors Through Cognitive Behavior Therapy and Task-Oriented Training. Stroke.

[92] Chon J., Soh Y., Shim G.Y. (2024). Stroke-Related Sarcopenia: Pathophysiology and Diagnostic Tools. Brain Neurorehabil..

[93] Nandy S., Parsons S., Cryer C., Underwood M., Rashbrook E., Carter Y., Eldridge S., Close J., Skelton D., Taylor S. (2004). Development and Preliminary Examination of the Predictive Validity of the Falls Risk Assessment Tool (FRAT) for Use in Primary Care. J. Public Health.

[94] Kempen G.I., Yardley L., van Haastregt J.C., Zijlstra G.A., Beyer N., Hauer K., Todd C. (2008). The Short FES-I: A Shortened Version of the Falls Efficacy Scale-International to Assess Fear of Falling. Age Ageing.

[95] Niedermann K., Meichtry A., Zindel B., Ernst M.J., Krafft V., Mattli R., Nast I., Wieser S., Wirz M., Brunner B. (2024). Effectiveness and Cost-Effectiveness of a Single Home-Based Fall Prevention Program: A Prospective Observational Study Based on Questionnaires and Claims Data. BMC Geriatr..

[96] Intaruk R., Saengsuwan J., Amatachaya S., Gaogasigam C., Thaweewannakij T. (2021). The Ability of Timed-Up and Go Test and Five Times Sit-to-Stand Test to Screen Risk of Fall in Well-Functioning Elderly. Health Sci. Tech. Rev..

[97] Cowey E., Schichtel M., Cheyne J.D., Tweedie L., Lehman R., Melifonwu R., Mead G.E. (2021). Palliative care after stroke: A review. Int. J. Stroke.

[98] Dyer S.M., Kwok W.S., Suen J., Dawson R., Kneale D., Sutcliffe K., Seppala L.J., Hill K.D., Kerse N., Murray G.R. (2025). Interventions for Preventing Falls in Older People in Care Facilities. Cochrane Database Syst. Rev..

[99] Medina-Rincón A., Pérez L.M., Bagur-Calafat C., Barrios-Franquesa A.M., Amor-Barbosa M., Doménech-García V., Bellosta-López P., Buesa-Estéllez A., Girabent-Farrés M. (2025). The Effect of Brief, Repetitive Balance Training on Balance and Fall Risk in Older People with Stroke: A Randomized Controlled Trial. Clin. Rehabil..

[100] Fujita K., Kobayashi Y., Miaki H., Hori H., Tsushima Y., Sakai R., Nomura T., Ogawa T., Kinoshita H., Nishida T. (2020). Pedaling Improves Gait Ability of Hemiparetic Patients with Stiff-Knee Gait: Fall Prevention during Gait. J. Stroke Cerebrovasc. Dis..

[101] Pigman J., Reisman D.S., Pohlig R.T., Wright T.R., Crenshaw J.R. (2019). The Development and Feasibility of Treadmill-Induced Fall Recovery Training Applied to Individuals with Chronic Stroke. BMC Neurol..

[102] Haley M.N., Sherrington C., Lawler K., Harding K.E., Lord M., Williams S., Taylor N.F. (2025). Falling Short on Implementation of Fall Prevention Guidelines in Health Services: A Systematic Review with Meta-Analysis. Age Ageing.

[103] Kotlęga D. (2025). Use of End-of-Life Care Pathways in Hospitalized Stroke Patients: A Retrospective Study of the AMBER Care and Dying Adults in the Last Days of Life Approaches. Healthcare.

[104] Eastman P., McCarthy G., Brand C.A., Weir L., Gorelik A., Le B. (2013). Who, Why and When: Stroke Care Unit Patients Seen by a Palliative Care Service within a Large Metropolitan Teaching Hospital. BMJ Support. Palliat. Care.

[105] Comer A.R., Williams L.S., Bartlett S., D’Cruz L., Endris K., Marchand M., Zepeda I., Toor S., Waite C., Jawed A. (2022). Palliative and End-of-Life Care after Severe Stroke. J. Pain Symptom Manag..

[106] Doubal F., Cowey E., Bailey F., Murray S.A., Borthwick S., Somerville M., Lerpiniere C., Reid L., Boyd K., Hynd G. (2018). The key challenges of discussing end-of-life stroke care with patients and families: A mixed-methods electronic survey of hospital and community healthcare professionals. J. R. Coll. Physicians Edinb..

[107] Zhang H., Davies C., Stokes D., O’Donnell D. (2025). Shared Decision-Making for Patients with Stroke in Neurocritical Care: A Qualitative Meta-Synthesis. Neurocrit. Care.

[108] Creutzfeldt C.J., Bu J., Comer A., Enguidanos S., Lutz B., Robinson M.T., Zahuranec D.B., Holloway R.G., On behalf of the American Heart Association Stroke Council, Council on Cardiovascular and Stroke Nursing, and Council on Clinical Cardiology (2025). Palliative and End-of-Life Care in Stroke: A Scientific Statement from the American Heart Association. Stroke.

[109] Ramsburg H., Moriarty H.J., MacKenzie Greenle M. (2024). End-of-Life Symptoms in Adult Patients With Stroke in the Last Two Years of Life: An Integrative Review. Am. J. Hosp. Palliat. Care.

[110] Liampas A., Velidakis N., Georgiou T., Vadalouca A., Varrassi G., Hadjigeorgiou G.M., Tsivgoulis G., Zis P. (2020). Prevalence and Management Challenges in Central Post-Stroke Neuropathic Pain: A Systematic Review and Meta-Analysis. Adv. Ther..

[111] Holloway R.G., Arnold R.M., Creutzfeldt C.J., Lewis E.F., Lutz B.J., McCann R.M., Rabinstein A.A., Saposnik G., Sheth K.N., Zahuranec D.B. (2014). American Heart Association Stroke Council; Council on Cardiovascular and Stroke Nursing; Council on Clinical Cardiology. Palliative and End-of-Life Care in Stroke: A Statement for Healthcare Professionals from the American Heart Association/American Stroke Association. Stroke.

[112] National Institute for Health and Care Excellence (NICE) (2019). End of Life Care for Adults: Service Delivery.

[113] Koffman J., Yorganci E., Murtagh F., Yi D., Gao W., Barclay S., Pickles A., Higginson I., Johnson H., Wilson R. (2019). The AMBER Care Bundle for Hospital Inpatients with Uncertain Recovery Nearing the End of Life: The Improve Care Feasibility Cluster RCT. Health Technol. Assess..

[114] Koffman J., Yorganci E., Yi D., Murtagh F.E.M., Gao W., Barclay S., Pickles A., Higginson I.J., Johnson H., Wilson R. (2019). Managing Uncertain Recovery for Patients Nearing the End of Life in Hospital: A Mixed-Methods Feasibility Cluster Randomised Controlled Trial of the AMBER Care Bundle. Trials.

[115] National Institute for Health and Care Excellence (NICE) (2015). Care of Dying Adults in the Last Days of Life.

[116] Liu Y., Kline D., Aerts S., Youngwerth J.M., Kutner J.S., Sillau S., Kluger B.M. (2017). Inpatient Palliative Care for Neurological Disorders: Lessons from a Large Retrospective Series. J. Palliat. Med..

[117] Steigleder T., Kollmar R., Ostgathe C. (2019). Palliative Care for Stroke Patients and Their Families: Barriers for Implementation. Front. Neurol..

[118] Govind N., Ferguson C., Phillips J.L., Hickman L. (2023). Palliative Care Interventions and End-of-Life Care as Reported by Patients Post-Stroke and Their Families: A Systematic Review. Eur. J. Cardiovasc. Nurs..

[119] Boone-Sautter K.M., Peterson M.A., Vermeesch K., Ahmed A. (2025). Palliative Care for Stroke Patients: Meeting the Quadruple Aim. J. Stroke Cerebrovasc. Dis..

[120] Lightbody C.E., Gordon C., Burton C., Davidson C., Jenkinson D., Patel A.S., Petrie F.J., Rouncefield-Swales A., Sprigg N., Stewart K. (2025). Prepare: Improving End-of-Life Care Practice in Stroke Care: Insights from a National Survey and Semi-Structured Interviews. Healthcare.

[121] Patel A., Berdunov V., Quayyum Z., King D., Knapp M., Wittenberg R. (2020). Estimated societal costs of stroke in the UK based on a discrete event simulation. Age Ageing.

[122] Lucas-Noll J., Clua-Espuny J.L., Lleixà-Fortuño M., Gavaldà-Espelta E., Queralt-Tomas L., Panisello-Tafalla A., Carles-Lavila M. (2023). The costs associated with stroke care continuum: A systematic review. Health Econ. Rev..

[123] Chakrabarty J., Naik A. (2024). A Hospital-Based Study to Assess Caregiver’s Burden among Caregivers of Stroke Survivors. Med. J. Dr. D.Y. Patil. Vidyapeeth.

[124] Kwon B.M., Lee H.H., Sohn M.K., Kim D.Y., Shin Y.-I., Oh G.-J., Lee Y.-S., Joo M.C., Lee S.Y., Song M.-K. (2023). Contributing Factors to the Burden on Primary Family Caregivers of Stroke Survivors in South Korea. Int. J. Environ. Res. Public Health.

[125] Gebremariam B., Mekie Y.M., Yihalem B., Yisak G., Tesfa K., Awoke A.Y., Gedlu N.S., Azeze E.G. (2023). Caregiver Burden and Its Associated Factors among Primary Caregivers of Stroke Survivors at Amhara Regional State Tertiary Hospitals: A Multicenter Study. Front. Stroke.

[126] Eriku G.A., Bekele G., Yitayal M.M., Belete Y., Girma Y. (2023). Depressive Symptoms and Its Associated Factors among Primary Caregivers of Stroke Survivors at Amhara Regional State Tertiary Hospitals: Multicenter Study. Neuropsychiatr. Dis. Treat..

[127] Joo H., Wang G., Yee S.L., Zhang P., Sleet D. (2017). Economic Burden of Informal Caregiving Associated with History of Stroke and Falls among Older Adults in the U.S.. Am. J. Prev. Med..

[128] Walsh M.E., Sorensen J., Galvin R., Williams D.J., Harbison J.A., Murphy S., Collins R., McCabe D.J., Crowe M., Horgan N.F. (2018). First Year Post-Stroke Healthcare Costs and Fall-Status among Those Discharged to the Community. Eur. Stroke J..

[129] Davis J.C., Robertson M.C., Ashe M.C., Liu-Ambrose T., Khan K.M., Marra C.A. (2010). International Comparison of Cost of Falls in Older Adults Living in the Community: A Systematic Review. Osteoporos. Int..

[130] Davis J.C., Dian L., Parmar N., Madden K., Khan K.M., Chan W., Cheung W., Rogers J., Liu-Ambrose T. (2018). Geriatrician-Led Evidence-Based Falls Prevention Clinic: A Prospective 12-Month Feasibility and Acceptability Cohort Study among Older Adults. BMJ Open.

[131] Novitzke J.M. (2008). The Rising Cost of Falling: Strategies to Combat a Common Post-Stroke Foe. J. Vasc. Interv. Neurol..

[132] Goldsmith S., Kokolakakis T. (2021). A Cost-Effectiveness Evaluation of Dance to Health: A Dance-Based Falls Prevention Exercise Programme in England. Public Health.

[133] Orman Z., Olaiya M.T., Thrift A.G., Cadilhac D.A., Phan T., Nelson M.R., Ung D., Srikanth V.K., Bladin C.F., Gerraty R.P. (2024). Cost-Effectiveness of an Individualised Management Program after Stroke: A Trial-Based Economic Evaluation. Neuroepidemiology.

[134] Wong A.K.C., Wang S.L., So C., Lian J., Yan Y., Li H., Wu L., Pei H., Wang W., Wong F.K.Y. (2024). Economic Evaluation of an Enhanced Post-Discharge Home-Based Care Program for Stroke Survivors. Value Health.

[135] Barbosa P.M., Szrek H., Ferreira L.N., Cruz V.T., Firmino-Machado J. (2024). Stroke Rehabilitation Pathways during the First Year: A Cost-Effectiveness Analysis from a Cohort of 460 Individuals. Ann. Phys. Rehabil. Med..

[136] Candio P., Violato M., Luengo-Fernandez R., Leal J. (2022). Cost-Effectiveness of Home-Based Stroke Rehabilitation across Europe: A Modelling Study. Health Policy.

[137] Tung Y.J., Lin W.C., Lee L.F., Lin H.M., Ho C.H., Chou W. (2021). Comparison of Cost-Effectiveness between Inpatient and Home-Based Post-Acute Care Models for Stroke Rehabilitation in Taiwan. Int. J. Environ. Res. Public Health.

[138] Rodgers H., Howel D., Bhattarai N., Cant R., Drummond A., Ford G.A., Forster A., Francis R., Hills K., Laverty A.M. (2019). Evaluation of an Extended Stroke Rehabilitation Service (EXTRAS): A Randomized Controlled Trial and Economic Analysis. Stroke.

[139] Pisavadia K., Anthony B.F., Davies J., Roberts S., Granger R., Spencer L.H., Gillen E., Hounsome J., Noyes J., Fitzsimmons D. (2024). The Cost-Effectiveness of Life after Stroke Services and the Impact of These Services on Health and Social Care Resource Use: A Rapid Review. medRxiv.

[140] Sharrief A.Z., Guzik A.K., Jones E., Okpala M., Love M.F., Ranasinghe T.I.J., Bushnell C. (2023). Telehealth Trials to Address Health Equity in Stroke Survivors. Stroke.

[141] Fujii R., Miki T., Nonaka Y., Tanaka S. (2024). Effectiveness of Telerehabilitation Based on Real-Time Intervention between Therapist and Participants for Improving Physical Function, Activities of Daily Living and Quality of Life in People with Stroke: A Systematic Review Protocol. PLoS ONE.

[142] Park H.Y., Yeom I.S., Kim Y.J. (2023). Telehealth Interventions to Support Self-Care of Stroke Survivors: An Integrative Review. Heliyon.

[143] Lawson D.W., Stolwyk R.J., Ponsford J.L., McKenzie D.P., Downing M.G., Wong D. (2020). Telehealth Delivery of Memory Rehabilitation Following Stroke. J. Int. Neuropsychol. Soc..

[144] Morrell K., Hyers M., Stuchiner T., Lucas L., Schwartz K., Mako J., Spinelli K.J., Yanase L. (2017). Telehealth Stroke Dysphagia Evaluation Is Safe and Effective. Cerebrovasc. Dis..

[145] Liang Q., Tao Y., He J., Bo Y., Xu L., Zhao F. (2024). Effects of Home-Based Telemedicine and mHealth Interventions on Blood Pressure in Stroke Patients: A Systematic Evaluation and Meta-Analysis of Randomized Controlled Trials. J. Stroke Cerebrovasc. Dis..

[146] Lam S.K.Y., Chau J.P.C., Lo S.H.S., Choi K.C., Siow E.K.C., Shum E.W.C., Lee V.W.Y., Hung S.S., Mok V.C.T., Ching J.Y.L. (2024). Evaluation of Cost-Effectiveness of a Virtual Multidisciplinary Stroke Care Clinic for Community-Dwelling Survivors of Stroke. J. Am. Heart Assoc..

[147] English C., Ramage E.R., Attia J., Bernhardt J., Bonevski B., Burke M., Galloway M., Hankey G.J., Janssen H., Lindley R. (2024). Secondary Prevention of Stroke: A Telehealth-Delivered Physical Activity and Diet Pilot Randomized Trial (ENAbLE-Pilot). Int. J. Stroke.

[148] Mohammadi F., Ardalan H.B., Dehghankar L., Motalebi S.A. (2023). Effect of Telenursing on the Quality of Life of Caregivers of Older Patients with Stroke. Rev. Recent. Clin. Trials.

[149] Walters K., Frost R., Avgerinou C., Kalwarowsky S., Goodman C., Clegg A., Marston L., Pan S., Hopkins J., Jowett C. (2025). Clinical and Cost-Effectiveness of a Home-Based Health Promotion Intervention for Older People with Mild Frailty in England: A Multicentre, Parallel-Group, Randomised Controlled Trial. Lancet Healthy Longev..

[150] Maheta B., Kraft A., Interrante N., Fereydooni S., Bailenson J., Beams B., Keny C., Osborne T., Giannitrapani K., Lorenz K. (2025). Using Virtual Reality to Improve Outcomes Related to Quality of Life among Older Adults with Serious Illnesses: Systematic Review of Randomized Controlled Trials. J. Med. Internet Res..

[151] Gan L., Huang L., Li L., Yang X., Meng L., Pang Z., Wei Q. (2025). ORLA Combined with Telerehabilitation in Patients with Subacute Poststroke Aphasia: A Randomized Controlled Trial. Sci. Rep..

[152] Sun S., Li Y., Zhang G., Zhang Y., Dong J. (2025). A Randomized Controlled Trial of Telerehabilitation Intervention for Acute Ischemic Stroke Patients Post-Discharge. J. Clin. Neurosci..

[153] Chen J., Sun D., Zhang S., Shi Y., Qiao F., Zhou Y., Liu J., Ren C. (2020). Effects of Home-Based Telerehabilitation in Patients with Stroke: A Randomized Controlled Trial. Neurology.

[154] Thielman G., Roos M. (2025). A Telehealth Exercise Program to Improve Cognition in People with Stroke. Sci. Rep..

[155] Butcher T., Warland A., Stewart V., Aweid B., Samiyappan A., Kal E., Ryan J., Athanasiou D.A., Baker K., Singla-Buxarrais G. (2025). Rehabilitation Using Virtual Gaming for Hospital and Home-Based Training for the Upper Limb in Acute and Subacute Stroke (RHOMBUS II): Results of a Feasibility Randomised Controlled Trial. BMJ Open.

[156] Kannan L., Vora J., Bhatt T., Hughes S.L. (2019). Cognitive-motor exergaming for reducing fall risk in people with chronic stroke: A randomized controlled trial. NeuroRehabilitation.

[157] Önal B., Köse N., Önal Ş.N., Zengin H.Y. (2025). Validity and Reliability of the Berg Balance Scale in Different Tele-Assessment Methods in Patients with Stroke. J. Eval. Clin. Pract..

[158] Grau-Pellicer M., Lalanza J.F., Jovell-Fernández E., Capdevila L. (2020). Impact of mHealth Technology on Adherence to Healthy Physical Activity after Stroke: A Randomized Study. Top. Stroke Rehabil..

[159] Aigbonoga D., Adewale B., Igwilo J., Adeyeye V., Olajide T., Olaniran O., Akintayo A., Aremu P., Oluwadamilare F., Popoola O. (2025). Efficacy of Short Message Service (SMS) Intervention on Medication Adherence and Knowledge of Stroke Prevention among Clinic Attendees at Risk of Stroke: A Randomized Controlled Trial. BMC Public Health.

[160] Pedreira da Fonseca E., da Silva Ribeiro N.M., Pinto E.B. (2017). Therapeutic Effect of Virtual Reality on Post-Stroke Patients: Randomized Clinical Trial. J. Stroke Cerebrovasc. Dis..

[161] Laver K.E., Lange B., George S., Deutsch J.E., Saposnik G., Chapman M., Crotty M. (2025). Virtual Reality for Stroke Rehabilitation. Cochrane Database Syst. Rev..

[162] Chumbler N.R., Li X., Quigley P., Morey M.C., Rose D., Griffiths P., Sanford J., Hoenig H. (2015). A Randomized Controlled Trial on Stroke Telerehabilitation: The Effects on Falls Self-Efficacy and Satisfaction with Care. J. Telemed. Telecare.

[163] Alwadai B., Lazem H., Almoajil H., Hall A.J., Mansoubi M., Dawes H. (2024). Telerehabilitation and Its Impact Following Stroke: An Umbrella Review of Systematic Reviews. J. Clin. Med..

[164] Laver K.E., Adey-Wakeling Z., Crotty M., Lannin N.A., George S., Sherrington C. (2020). Telerehabilitation Services for Stroke. Cochrane Database Syst. Rev..

[165] Yang B., Gong E., Chen X., Tan J., Peoples N., Li Y., Cai J., Li Y., Oldenburg B., Chen C. (2025). Economic Evaluation of a Multicomponent mHealth Intervention for Stroke Management in Rural China: Cluster-Randomized Trial with 6-Year Follow-Up. JMIR Mhealth Uhealth.

[166] Shnitzer H., Chan J., Yau T., McIntyre M., Andreoli A., Kua A., Bayley M., Leochico C.F., Guo M., Munce S. (2025). The Safety of Telerehabilitation: Systematic Review. JMIR Rehabil. Assist. Technol..

[167] Verma A., Towfighi A., Brown A., Abhat A., Casillas A. (2022). Moving Towards Equity with Digital Health Innovations for Stroke Care. Stroke.

[168] Hepburn J., Williams L., McCann L. (2025). Barriers to and Facilitators of Digital Health Technology Adoption among Older Adults with Chronic Diseases: Updated Systematic Review. JMIR Aging.

[169] Niyomyart A., Ruksakulpiwat S., Benjasirisan C., Phianhasin L., Nigussie K., Thorngthip S., Shamita G., Thampakkul J., Begashaw L. (2024). Current Status of Barriers to mHealth Access among Patients with Stroke and Steps toward the Digital Health Era: Systematic Review. JMIR Mhealth Uhealth.

[170] Haimi M. (2023). The Tragic Paradoxical Effect of Telemedicine on Healthcare Disparities—A Time for Redemption: A Narrative Review. BMC Med. Inform. Decis. Mak..

[171] Chen Y., Chen Y., Zheng K., Dodakian L., See J., Zhou R., Chiu N., Augsburger R., McKenzie A., Cramer S.C. (2020). A Qualitative Study on User Acceptance of a Home-Based Stroke Telerehabilitation System. Top. Stroke Rehabil..

[172] Söderhielm K., Tistad M., Ytterberg C., Guidetti S. (2025). Experiences of F@ce 2.0: A Person-Centred Intervention for Home-Based Rehabilitation after Stroke Supported by Digital Technology—A Qualitative Study. BMJ Open.

[173] Oliveira L.C., Dos Santos H.M., da Silva M.A., de Oliveira B.S.L., de Lima T.S., Pereira G.S., Silva S.M. (2025). Tele-Assessment of Activities and Participation in the Chronic Phase of Stroke: Is Use Valid and Viable in a Developing Country?. J. Telemed. Telecare.

[174] Ogedegbe G., Teresi J.A., Williams S.K., Ogunlade A., Izeogu C., Eimicke J.P., Kong J., Silver S.A., Williams O., Valsamis H. (2024). Home Blood Pressure Telemonitoring and Nurse Case Management in Black and Hispanic Patients with Stroke: A Randomized Clinical Trial. JAMA.

